# RETRACTED: Alminderej et al. Antimicrobial and Wound Healing Potential of a New Chemotype from *Piper cubeba* L. Essential Oil and In Silico Study on *S. aureus* tyrosyl-tRNA Synthetase Protein. *Plants* 2021, *10*, 205

**DOI:** 10.3390/plants13030334

**Published:** 2024-01-23

**Authors:** Fahad Alminderej, Sana Bakari, Tariq I. Almundarij, Mejdi Snoussi, Kaïss Aouadi, Adel Kadri

**Affiliations:** 1 Department of Chemistry, College of Science, Qassim University, Buraidah 51452, Saudi Arabia; f.alminderej@qu.edu.sa (F.A.); k.aouadi@qu.edu.sa (K.A.); 2 Department of Chemistry, Faculty of Science of Sfax, University of Sfax, B.P. 1171, Sfax 3000, Tunisia; sana.bakari@yahoo.fr; 3 Department of Veterinary Medicine, College of Agriculture and Veterinary Medicine, Qassim University, P.O. Box 6622, Buraidah 51452, Saudi Arabia; tmndrj@qu.edu.sa; 4 Department of Biology, College of Science, Hail University, P.O. Box 2440, Ha’il 2440, Saudi Arabia; snmejdi@gmail.com; 5 Laboratory of Genetics, Biodiversity and Valorization of Bio-Resources (LR11ES41), University of Monastir, Higher Institute of Biotechnology of Monastir, Avenue Tahar Haddad, BP74, Monastir 5000, Tunisia; 6 Faculty of Sciences of Monastir, University of Monastir, Avenue of the Environment, Monastir 5019, Tunisia; 7 Faculty of Science and Arts in Baljurashi, Albaha University, P.O. Box (1988), Albaha 65527, Saudi Arabia

The journal retracts the article, ‘Antimicrobial and Wound Healing Potential of a New Chemotype from *Piper cubeba* L. Essential Oil and In Silico Study on *S. aureus* tyrosyl-tRNA Synthetase Protein’ [1].

Following publication, concerns were brought to the attention of the publisher regarding the overlapping figures across three other publications [2,3,4]. More specifically, the subfigure Group 4-Day 5 of Figure 3 in the paper [1] is duplicated as the subfigure Group II and III-Day 7 of Figure 5 in the published paper [2], and subfigure day 8, EPS22 hydrogel of Figure 4A in the paper [3]. The subfigure Group 2-Day 3 is duplicated in two papers as the subfigures CICAFLORA treated group-day 4 in Figure 4a [3], and Reference group, Day 3 in Figure 2 [4]. The subfigure Group 1-Day 5 is also duplicated in two papers as the subfigures CICAFLORA treated group-day 6 in Figure 4a [3], and the Reference group, Day 7 in Figure 2 [4]. The subfigure Group 1-Day 7 is duplicated as the untreated group-day 10 of Figure 4 in the article [3] and the Untreated group, Day 11 in Figure 2 [4]. The subfigures in Figure 3 of the paper [1] also shows image manipulation within the figure, Group 1-Day 9 is duplicated with Group 2-Day 9, Group 1-Day 11 is duplicated with Group 4-Day 7 and four subfigures in Day 1 are the same. The Day 1 subfigures are also duplicated as Day 1 in Figure 2 of [4].

Adhering to our complaint’s procedure, an investigation was conducted by the Editorial Office and Editorial Board that confirmed the overlap of the figures across these four publications [1,2,3,4]. Consequently, the Editorial Office and the Editorial Board no longer have confidence in the findings and have decided to retract this article [1] as per MDPI’s retraction policy (https://www.mdpi.com/ethics#_bookmark30).

This retraction was approved by the Editor-in-Chief of the journal *Plants*.

The authors did not agree to this retraction.

## References

[1] Alminderej F., Bakari S., Almundarij T.I., Snoussi M., Aouadi K., Kadri A. (2021). Antimicrobial and Wound Healing Potential of a New Chemotype from *Piper cubeba* L. Essential Oil and In Silico Study on *S. aureus* tyrosyl-tRNA Synthetase Protein. Plants.

[2] Ben Ayed H., Bardaa S., Moalla D., Jridi M., Maalej H., Sahnoun Z., Rebai T., Jacques P., Nasri M., Hmidet N. (2015). Wound healing and in vitro antioxidant activities of lipopeptides mixture produced by *Bacillus mojavensis* A21. Process Biochem..

[3] Maalej H., Moalla D., Boisset C., Bardaa S., Ben Ayed H., Sahnoun Z., Rebai T., Nasri M., Hmidet N. (2014). Rhelogical, dermal wound healing and in vitro antioxidant properties of exopolysaccharide hydrogel from *Pseudomonas stutzeri* AS22. Colloids Surf. B Biointerfaces.

[4] Bardaa S., Ben Halima N., Aloui F., Ben Mansour R., Jabeur H., Bouaziz M., Sahnoun Z. (2016). Oil from pumpkin (*Cucurbita pepo* L.) seeds: Evaluation of its functional properties on wound healing in rats. Lipids Health Dis..

